# Retraction Note: Can Two-Way Direct Communication Protocols Be Considered Secure?

**DOI:** 10.1186/s11671-019-3086-8

**Published:** 2019-07-18

**Authors:** Springer Science+Business Media, LLC

**Retraction Note: Nanoscale Res Lett (2017) 12:552**

**https://doi.org/10.1186/s11671-017-2314-3**

The editors have retracted this article [1] because after publication concerns were raised regarding the validity of the conclusions drawn. Post-publication peer review has revealed a flaw in the application of the key rate equation r = IAB-IAE. For calculation of the term IAE, the effect of disturbance (D) on both the message mode (MM) and control mode (CM) was not taken into account. The main claim of the paper cannot be reliably reached. The author does not agree to this retraction.
